# Identification of Two m6A Readers *YTHDF1* and *IGF2BP2* as Immune Biomarkers in Head and Neck Squamous Cell Carcinoma

**DOI:** 10.3389/fgene.2022.903634

**Published:** 2022-05-12

**Authors:** Shaojie Li, Qiuji Wu, Jia Liu, Yahua Zhong

**Affiliations:** Hubei Key Laboratory of Tumor Biological Behaviors, Department of Radiation and Medical Oncology, Hubei Cancer Clinical Study Center, Zhongnan Hospital of Wuhan University, Wuhan, China

**Keywords:** YTHDF1, IGF2BP2, HNSCC, m6A modification, immune microenvironment, immunotherapy

## Abstract

**Background:** N6-methyladenosine (m6A) is the most abundant internal modification pattern in mammals that a plays critical role in tumorigenesis and immune regulations. However, the effect of m6A modification on head and neck squamous cell carcinoma (HNSCC) has not been clearly studied.

**Methods:** We screened m6A regulators that were significantly correlated with tumor immune status indicated by ImmuneScore using The Cancer Genome Atlas (TCGA) dataset and obtained distinct patient clusters based on the expression of these m6A regulators with the R package “CensusClusterPlus.” We then performed gene set enrichment analysis (GSEA), CIBERSORT, and single-sample gene set enrichment analysis (ssGSEA) to assess the differences in gene function enrichment and tumor immune microenvironment (TIME) among these clusters. We further conducted differently expressed gene (DEG) analysis and weighted gene co-expression network analysis (WGCNA) and constructed a protein–protein interaction (PPI) network to determine hub genes among these clusters. Finally, we used the GSE65858 dataset as an external validation cohort to confirm the immune profiles related to the expression of m6A regulators.

**Results:** Two m6A readers, *YTHDF1* and *IGF2BP2*, were found to be significantly associated with distinct immune status in HNSCC. Accordingly, patients were divided into two clusters with Cluster 1 showing high expression of *YTHDF1* and *IGF2BP2* and Cluster 2 showing low expression levels of both genes. Clinicopathologically, patients from Cluster 1 had more advanced T stage and pathological grades than those from Cluster 2. GSEA showed that Cluster 1 was closely related to the RNA modification process and Cluster 2 was significantly correlated with immune regulations. Cluster 2 had a more active TIME characterized by a more relative abundance of CD8^+^ T cells and CD4^+^ T cells and higher levels of MHC I and MHC II molecules. We constructed a PPI network composed of 16 hub genes between the two clusters, which participated in the T-cell receptor signaling pathway. These results were externally validated in the GSE65858 dataset.

**Conclusions:** The m6A readers, *YTHDF1* and *IGF2BP2,* were potential immune biomarkers in HNSCC and could be potential treatment targets for cancer immunotherapy.

## Introduction

M6A is a critical and abundant internal epigenetic modification on both messenger RNA (mRNA) and non-coding RNAs in mammals. M6A modification is mainly found in the 3′ untranslated regions of the RNA. The reversible and dynamic regulation of m6A is mainly mediated by three different kinds of regulators, namely, writers, erasers, and readers (Roundtree et al., 2017; Nombela et al., 2021). Writers and erasers are methyltransferases (such as METTL3, METTL16, and WTAP) and demethylases (such as ALKBH5 and FTO) that methylate and demethylate RNA adenosine at specific N6 positions, respectively. Readers are a group of RNA binding proteins that recognize m6A sites and initiate downstream events such as RNA splicing, maturation, degradation, and translation (Li Y et al., 2019; Zaccara et al., 2019). These proteins include members of the EIF3, IGF2BP family and YTH family. By modulating RNA export, RNA stability, protein expression, and other biological activities, m6A modification plays an essential role in cancer development. In line with these findings, emerging drugs targeting m6A modification, such as a selective inhibitor of FTO, METTL3, and YTHDF2 have shown promising anti-cancer effects (Huang et al., 2015; Visvanathan et al., 2018; Dixit et al., 2021).

The detailed mechanism by which m6A modification impacts cancer pathogenesis remains unclear. A recent study indicated that m6A also has a dual role in tumorigenesis. Liu et al. reported that YTHDF1 promoted ovarian cancer progression *via* augmenting EIF3C translation (Liu et al., 2020). Li et al. reported that IGF2BP2 prevented SOX2 degradation, leading to colorectal cancer pathogenesis and progression (Li T et al., 2019). Meanwhile, Zhong et al. reported that m6A helped suppress hepatocellular carcinoma through YTHDF2-directed degradation of EGFR (Zhong et al., 2019). Importantly, m6A modification also has a nonnegligible impact on anti-tumor immunity. Overexpression of YTHDF1 enhanced the stability of RNA lysosomal proteases, which led to the degradation of tumor antigens in dendritic cells, disabled CD8^+^ T cells to bring about immunosurveillance and abolished the effect of immune checkpoint inhibitors (ICIs) (Han et al., 2019). Suppression of METTL3 and METTL14 increased infiltration of CD8^+^ T cells and secretion of IFN-γ, CXCL9, and CXCL10 in TIME and promoted the response to ICIs in melanoma and pMMR-MSI^low^ colorectal cancer (Wang et al., 2020). However, research aiming to explore the effect of m6A modification on immune profiles in HNSCC is inadequate.

HNSCC is the sixth most common malignant tumor worldwide. Its occurrence is closely linked to carcinogen exposure and viral infection, especially human papillomavirus (HPV) and Epstein–Barr virus (EBV) (Siegel et al., 2021). HNSCC is a group of heterogeneous cancers, and the majority of patients are presented with locally advanced or metastatic stage, leading to poor prognosis (Argiris et al., 2008; Chow, 2020). ICIs-based immunotherapy has prominently improved the efficacy and survival of advanced HNSCC. However, only a small subset of patients could benefit from immunotherapy. Although a combined positive score (CPS) of PD-L1 expression is mostly used to guide immunotherapy, currently no satisfactory predictive biomarker is available for HNSCC.

Here, we attempted to explore the role of m6A regulators in the immune modulation of HNSCC and tried to identify potential m6A-associated biomarkers of immunotherapy in HNSCC. This study might provide a new way to improve the effect of immunotherapy in HNSCC.

## Methods and Materials

### Data Sources and Processing

We downloaded the transcriptomic data (HTSeq-FPKM and HTSeq-Counts) and clinical information of an HNSCC cohort from the TCGA-database (https://portal.gdc.cancer.gov/). Data of HTSeq-Counts was used to analyze DEGs, and HTSeq-FPKM was used to conduct ESTIMATE, clustering, CIBERSORT, ssGSEA, and WGCNA. Mann-Whitney *U* test was performed to compare age and gene expression between two groups. Chi-square test was utilized to compare gender, T stage, N stage, and pathologic stage. Spearman’s coefficient was used to conduct correlation analysis. And a *p*-value < 0.05 (two-sided) was considered statistically significant. Meanwhile, we conducted an external validation dataset by downloading the expression profiling data of the GSE65858 array from the Gene Expression Omnibus (GEO) database (https://www.ncbi.nlm.nih.gov/geo/).

### Estimation of Stromal and Immune Cells

We employed the ESTIMATE tool embedded in the R package “estimate” that used gene expression signatures to infer the fraction of stromal and immune cells in the tumor samples and to estimate the elements of tumor microenvironment (TME), including StromalScore, ImmuneScore, ESTIMATEScore, and TumorPurity (Yoshihara et al., 2013).

### Consensus Clustering

To explore the influence of m6A modification on immune profiles of HNSCC, we calculated the correlation between the expression of m6A modification regulators and ESTIMATE results with Spearman’s coefficient. And then we performed consensus clustering of tumor samples based on the expression of *YTHDF1* and *IGF2BP2*. We accomplished consensus clustering and result visualization with the R package “ConsensusClusterPlus” (Wilkerson and Hayes, 2010). And we examined the efficacy of the above consensus clustering by principal component analysis (PCA) with the R package “factoextra.” The Kaplan–Meier method and log-rank test were utilized to compare overall survival between the two clusters.

### Gene Set Enrichment Analysis

We employed the software GSEA (https://www.gsea-msigdb.org/gsea/) to determine different pathways enriched in the two clusters based on the default defined set of genes (Mootha et al., 2003; Subramanian et al., 2005). We selected “c5.go.cc.v7.4.symbols.gmt” from MSigDB Collection as pre-defined ontology gene set, and considered a pathway as significantly enriched pathway with the absolute normalized enrichment score > 1 (|NES| >1) and *p* value < 0.05.

### Immune Microenvironment Analysis

CIBERSORT, a method excelling in decreasing noise and unknown mixtures and identifying similar cell types, was conducted to recognize the cell composition of solid tumors by using gene expression profiles (Newman et al., 2015). ssGSEA, an extension of GSEA, was used to calculate separate enrichment scores for each pairing of a sample and gene set (Hänzelmann et al., 2013). We applied both strategies to explore the difference and relation of TIME among the clusters of patients with HNSCC.

### DEGs and Weighted Gene Co-Expression Network Analysis

In order to explore the hub genes that contributed to biological divergences among different patient clusters, we performed an analysis of DEGs and WGCNA successively. First, the R package “limma” enabled us to compare transcriptome data (HTSeq-Counts) to locate DEGs. The screening thresholds were set as |log2FoldChange | > 0.6 and *p*-value of < 0.05, and the results were visualized by volcano plot and heat map. Then, we conducted WGCNA on the DEGs with the R package “WGCNA,” which was a system biology method to gather closely related genes in special modules and calculate the relationship between the modules and external sample traits (Zhang and Horvath, 2005; Langfelder and Horvath, 2008). We explored the connection of DEGs with clustering and four aspects of ESTIMATE. Finally, we selected the module of genes tightly related to both clustering and ImmuneScore for subsequent analysis.

### Functional Enrichment and Protein–Protein Interaction Network Analysis

In order to investigate the above module of genes ulteriorly, we uploaded the gene list to Metascape (http://metascape.org/) for pathway and process enrichment analysis and PPI enrichment analysis (Zhou et al., 2019). Functional enrichment analysis was carried out in various ontology sources, including GO Biological Processes, Reactome Gene Sets, KEGG Pathway, Canonical Pathways, and WikiPathways. PPI enrichment analysis was also performed, and if the number of proteins in the network fell between 3 and 500, the Molecular Complex Detection (MCODE) algorithm would be carried out to separate proteins to build interaction networks more precisely (Bader and Hogue, 2003).

## Results

### Identification of m6A Regulators Associated With HNSCC Immune Profiles

**FIGURE 1:**
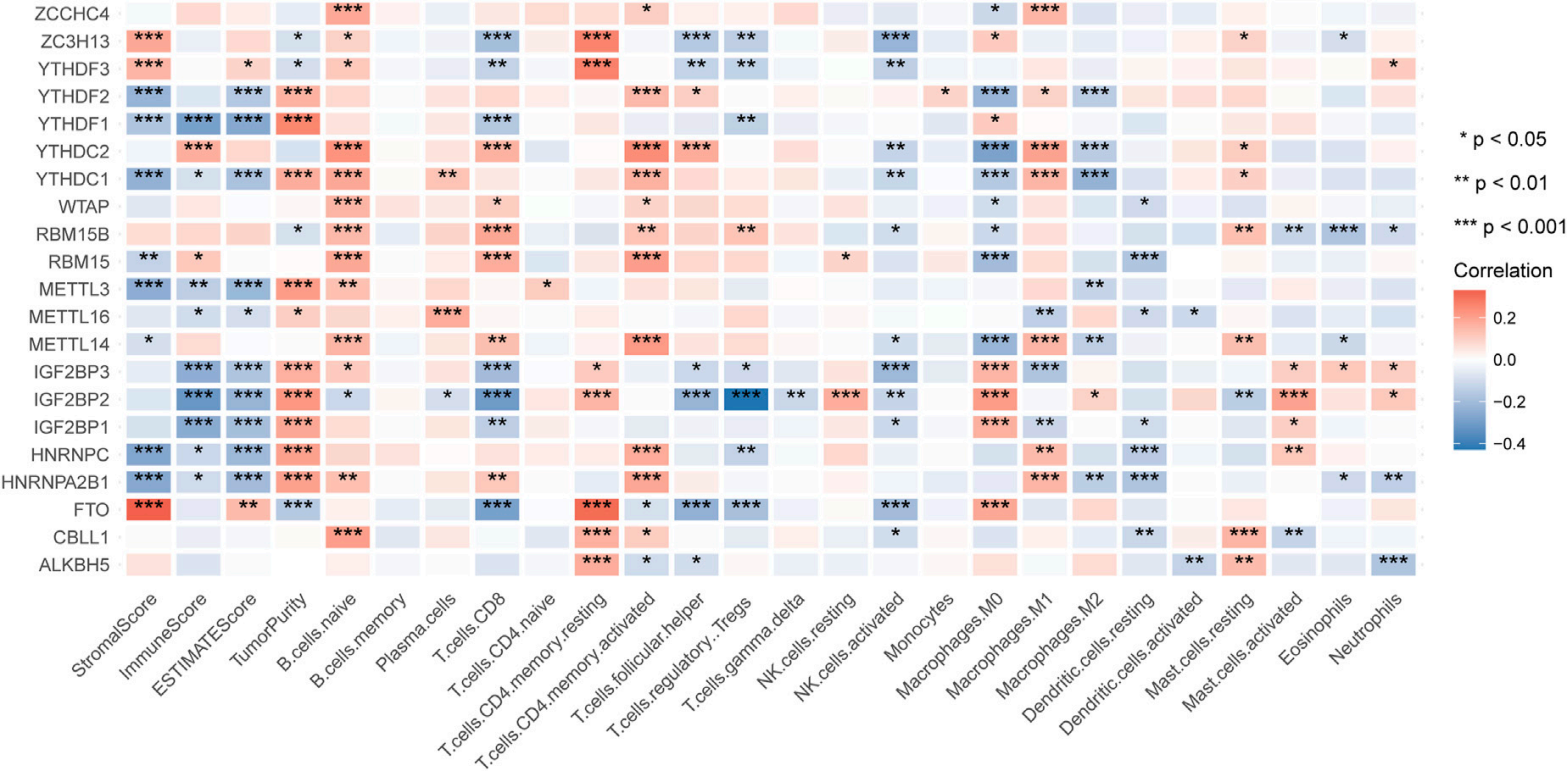
The correlation of m6A regulators with the results of ESTIMATE and CIBERSORT.

After excluding repeated samples and those without adequate survival information, we got 499 patients of HNSCC with unique samples for the following analysis. To explore whether the expression of m6A regulators impacted HNSCC immune profiles, we extracted the expression of 21 m6A modification regulators (Table 1) and applied the ESTIMATE tool and CIBERSORT algorithm to calculate the ESTIMATE scores and immune cells infiltration of 499 HNSCC patients. By analyzing the correlation between m6A regulators’ expression and the ImmuneScore, we found that *YTHDC2* and *RBM15* were positively correlated with ImmuneScore, while *YTHDF1*, *YTHDC1*, *METTL3*, *METTL16*, *IGF2BP1-3*, *HNRNPC,* and *HNRNPA2B1* were negatively correlated with ImmuneScore (Figure 1). Next, we sorted the absolute values of the ImmuneScores correlated with the 21 m6A regulators (Supplementary Material S1). We selected the first two regulators with the highest ImmuneScores, *YTHDF1* and *IGF2BP2*, to construct an immune-associated signature.

**TABLE 1:** Summary of common m6A regulators.

Type	Regulator	Function
Writer	CBLL1	Stabilizes several subunits of the methyltransferase complex
	METTL3	Catalyzes m6A modification
	METTL14	Provides help of target recognition for METTL3
	METTL16	Catalyzes m6A modification
	RBM15/RBM15B	Binds the m6A methylation complex and recruit it to specific sites in RNA
	WTAP	Helps localization of METTL3-METTL14 into nuclear speckles
	ZC3H13	Anchors WTAP, Virilizer, and Hakai in the nucleus to facilitate m6A methylation and regulate mESC self-renewal
	ZCCHC4	Catalyzes m6A modification of 28S ribosomal RNA
Eraser	ALKBH5	Removes m6A modification
	FTO	Removes m6A modification
Reader	EIF3	Promotes mRNA translation
	HNRNPA2B1	Mediates effects of m6A modification on primary microRNA processing and alternative splicing
	HNRNPC	Affects mRNA abundance and alternative splicing
	IGF2BPs	Enhances mRNA stability
	YTHDC1	Enhances RNA splicing and export
	YTHDC2	Promotes the translation of target RNA and reduce its abundance
	YTHDF1	Promotes mRNA translation
	YTHDF2	Promotes mRNA degradation
	YTHDF3	Interacts with YTHDF1 and YTHDF2 to enhance their effect

### Consensus Clustering of Patients With HNSCC Based on YTHDF1 and IGF2BP2

**FIGURE 2:**
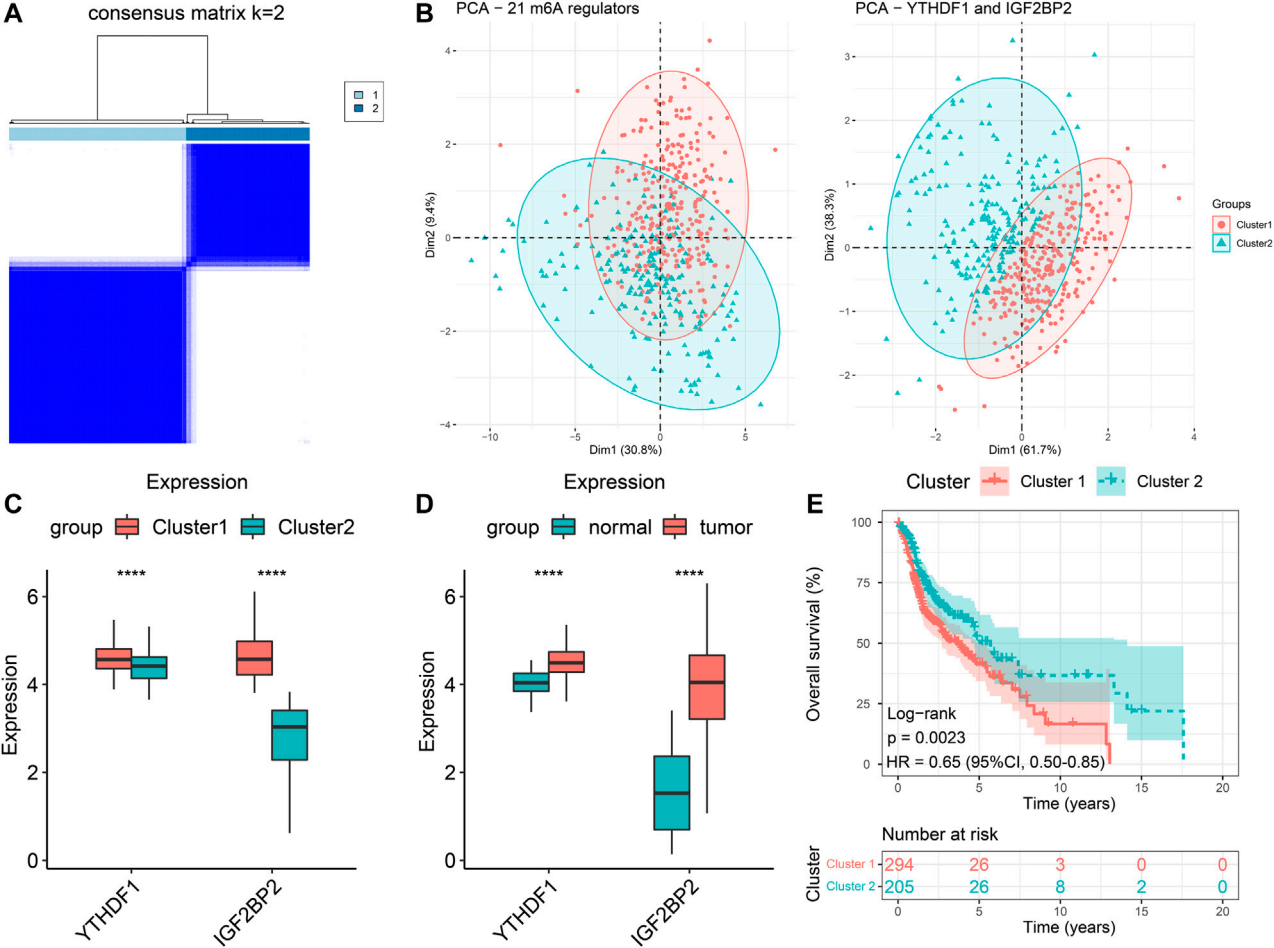
Clustering of patients with HNSCC in TCGA cohort based on expression of YTHDF1 and IGF2BP2. **(A)** Consensus clustering matrix for k = 2. **(B)** The results of PCA of clustering based on 21 m6A regulators as well as *YTHDF1* and *IGF2BP2*. **(C)** Comparison the expression levels of *YTHDF1* and *IGF2BP2* between Cluster 1 and Cluster 2. **(D)** Comparison of the expression levels of *YTHDF1* and *IGF2BP2* between tumor samples and normal samples. **(E)** Kaplan–Meier curves of the overall survival in two clusters. ****p < 0.0001.

We extracted the expression data of *YTHDF1* and *IGF2BP2* of the 499 HNSCC patients and performed consensus clustering, and obtained two clusters of patients (Figure 2A). There were 294 patients in Cluster 1 and 205 patients in Cluster 2. After excluding 106 patients without tumor stage and tumor grade information, the clinical characteristics of the remaining 393 patients were summarized in Table 2. PCA plot indicated the above clustering had good efficiency of distinction (Figure 2B). Cluster 1 had higher expression of *YTHDF1* and *IGF2BP2* (Figure 2C), and a more advanced T stage and pathological grade than Cluster 2 (Table 2). Expression levels of *YTHDF1* and *IGF2BP2* were also compared between tumor tissue and normal tissue, and we found that both of them were higher expressed in tumor tissue (Figure 2D). Kaplan–Meier curve (Figure 2E) showed that patients in Cluster 2 had better overall survival than their counterparts in Cluster 1 (HR = 0.65, 95%CI [0.50–0.85], *p* = 0.0023).

**TABLE 2:** Summary of clinical characteristics of patients in the two clusters.

	Cluster 1 (*n* = 245)	Cluster 2 (*n* = 148)	*p* value
Age, median (range)	60 (19–90)	61 (24–87)	0.28
Gender, *n* (%)			0.47
Female	63 (25.7)	43 (29.1)	—
Male	182 (74.3)	105 (70.9)	—
T stage, *n* (%)			0.0077
T1	18 (7.4)	23 (15.5)	—
T2	59 (24.1)	42 (28.4)	—
T3	66 (26.9)	23 (15.6)	—
T4	102 (41.6)	60 (40.5)	—
N stage, *n* (%)			0.50
N0	101 (41.2)	68 (45.9)	—
N1	39 (15.9)	24 (16.2)	—
N2	99 (40.4)	55 (37.2)	—
N3	6 (2.4)	1 (0.7)	—
Pathological stage, *n* (%)			0.14
Stage I	12 (4.9)	15 (10.1)	—
Stage II	30 (12.2)	19 (12.8)	—
Stage III	51 (20.8)	22 (14.9)	—
Stage IV	152 (62.0)	92 (62.2)	—
Grade, *n* (%)			0.029
G1	22 (9.0)	28 (18.9)	—
G2	162 (66.1)	84 (56.8)	—
G3	60 (24.5)	36 (24.3)	—
G4	1 (0.4)	0 (0)	—

### Immune Profiles of YTHDF1- and IGF2BP2-Based Clusters

**FIGURE 3:**
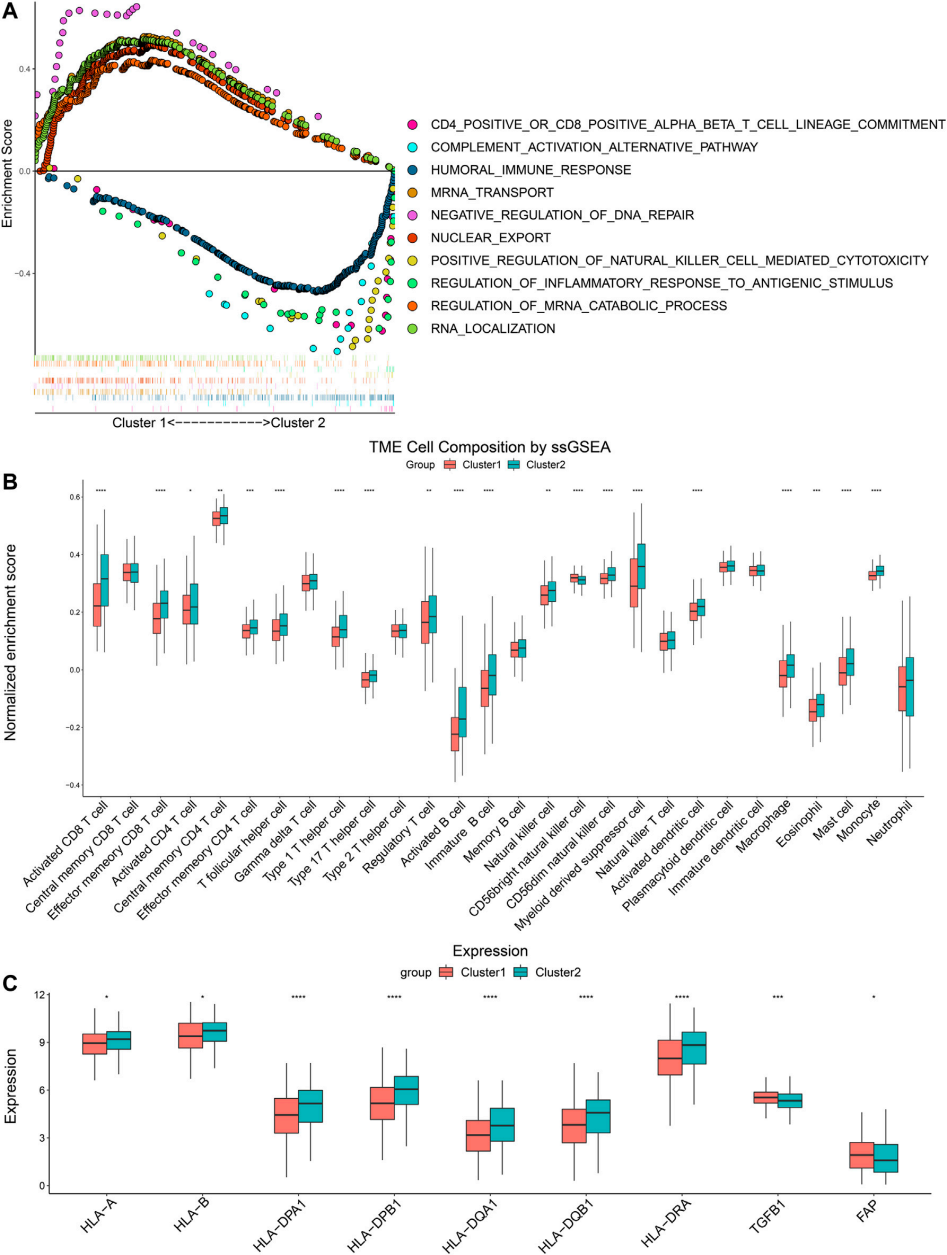
Differences of GSEA and immune cells infiltration between two clusters. **(A)** The tendency of enrichment of biological pathways between two clusters. **(B)** ssGSEA indicated different immune cells infiltration between two clusters. **(C)** Comparison of immune-related molecules between Cluster 1 and Cluster 2. *p < 0.05, **p < 0.01, ***p < 0.001, ****p < 0.0001.

GSEA was performed to compare pathway enrichment between the two clusters. We found that biological pathways related to m6A modification including negative regulation of DNA repair, regulation of mRNA catabolic progress, and nuclear export, were enriched in Cluster 1. On the other side, immune-related biological pathways, such as humoral immune response, positive regulation of NK cell-mediated cytotoxicity, and regulation of inflammatory response to an antigenic stimulus, were more enriched in Cluster 2 (Figure 3A). These results indicated that Cluster 2 was closely associated with immune modulation of head and neck cancers. In order to comprehend the difference in immune infiltration profiles between Cluster 1 and Cluster 2, we performed CIBERSORT, ssGSEA, and compared the expression of immune-related genes. The results of CIBERSORT indicated that CD4^+^ T memory resting cells, resting NK cells, M0 macrophages, and activated mast cells had higher percentages in Cluster 1. Cluster 2 highly expressed plasma cells, CD8^+^ T cells, regulatory T cells, and resting mast cells (Supplementary Figure S1). Furthermore, the results of ssGSEA were in parallel with the results of CIBERSORT and demonstrated the majority of immune cell types, including activated CD8^+^ T cells, activated CD4^+^ T cells, activated B cells, and natural killer cells, were enriched in the TIME of Cluster 2. Therefore, Cluster 2 manifested more active anti-tumor immune cell gathering (Figure 3B). Next, we compared the expression of critical immune-related molecules. Both MHC I and II molecules played a central role in the adaptive immune response. MHC I molecule were encoded by *HLA-A* and *HLA-B* genes, and MHC II molecules were encoded by *HLA-DP*, *HLA-DQ,* and *HLA-DR* genes. Cluster 2 had higher levels of MHC I and II molecules compared with Cluster 1. *TGFB1* encoded transforming growth factor-β (TGF-β) and *FAP* coded fibroblast activation protein alpha (FAP), both of which took part in disabling anti-tumor immune cells and impeding infiltration of immune cells. Cluster 2 had lower levels of TGF-β and FAP than Cluster 1 (Figure 3C). These results showed that Cluster 2 had a more immune-stimulatory TIME than Cluster 1.

## Association of YTHDF1- and IGF2BP2-Based Clustering With HNSCC-Related Genes

**FIGURE 4:**
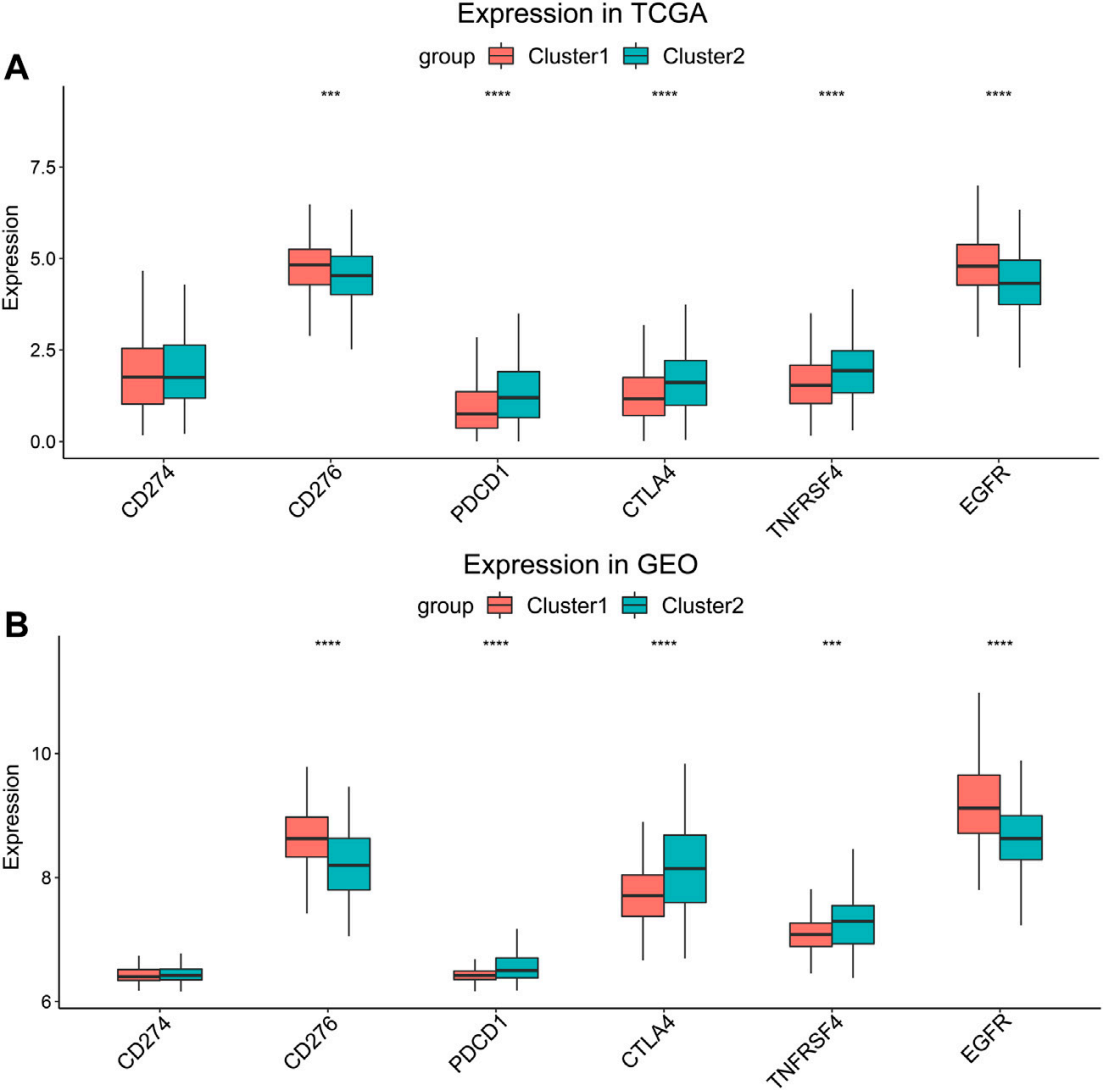
Expression level comparison of HNSCC-related genes. **(A)** TCGA cohort and **(B)** GEO cohort. ***p < 0.001, ****p < 0.0001.

Several genes were known to influence biological behavior and response to immunotherapy of HNSCC (Supplementary Material S2). We compared their expression levels between Cluster 1 and Cluster 2 in the TCGA cohort (Figure 4A) and the GEO cohort (Figure 4B). *PDCD1*, *CTLA4,* and *TNFRSF4* (encoding OX40) were higher expressed in Cluster 2, while the expression of *CD276* and *EGFR* were higher in Cluster 1. But there was no significant difference in the expression of *CD274* (encoding PD-L1) between the two clusters. The higher expression of *CD276* and *EGFR* suggested that the HNSCC of Cluster 1 might connect with worse biological behavior.

### Identifying Hub Genes by DEG Analysis and WGCNA

**FIGURE 5:**
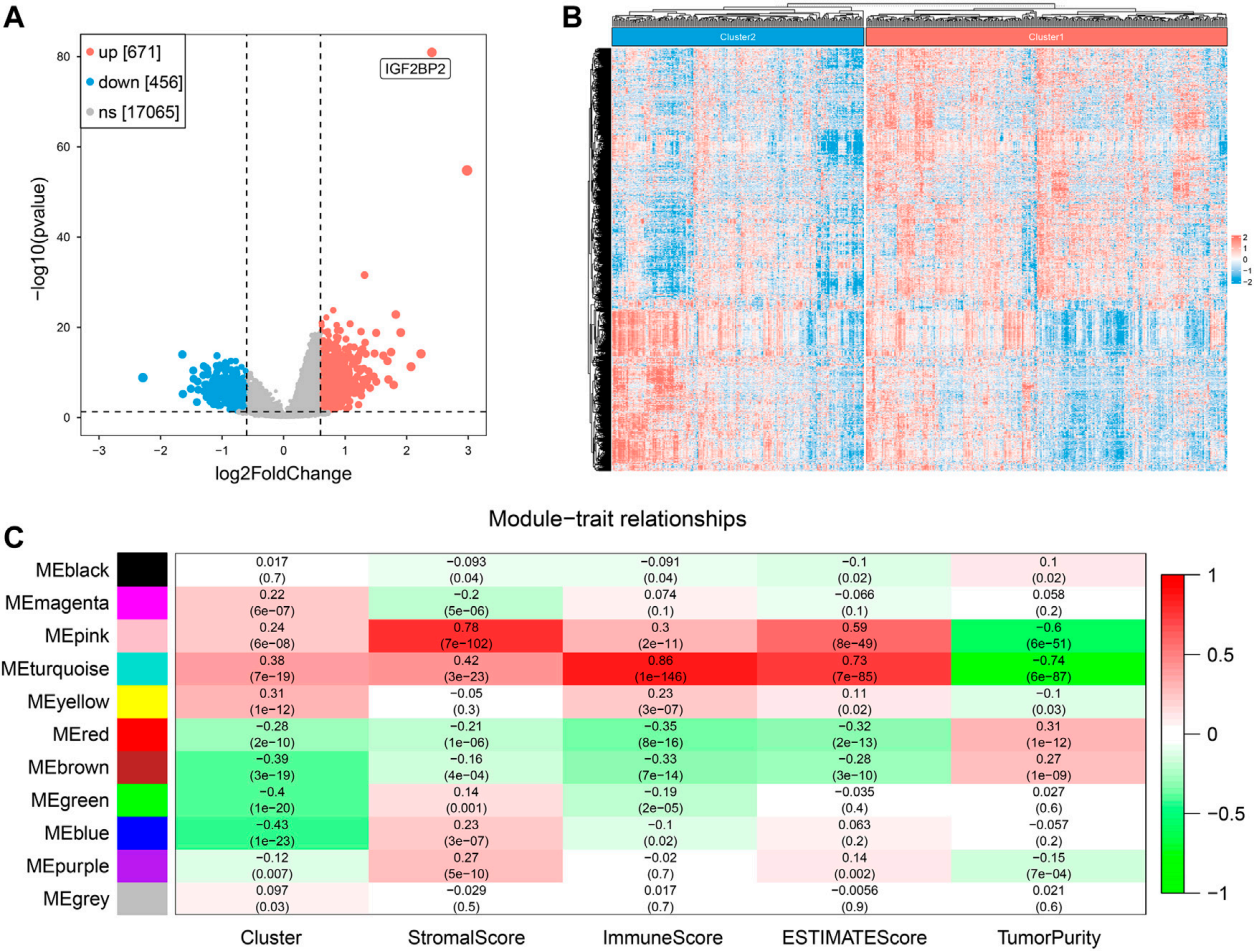
Screening of hub genes related to clustering and ImmuneScore in two clusters. **(A)** The volcano plot for results of differentially expressed genes. **(B)** The heatmap of differentially expressed genes. **(C)** Correlation of gene modules with results of clustering and ESTIMATE.

After removing duplicated samples and extracting mRNA expression from original transcriptome data (HTSeq-Counts), we got a matrix of 499 tumor patients with a unique sample and 18,192 gene expression data. We performed DEG analysis between the two clusters and obtained 1127 DEGs (671 upregulated and 456 downregulated). A volcano plot and heatmap were used to visualize the DEG results (Figures 5A,B). We set 3 as the soft power (Supplementary Figure S2) and then divided the 1127 DEGs into 11 modules by conducting WGCNA (Supplementary Figure S3), and we found that the turquoise module, containing 416 genes, was the most relevant with both clustering (*R* = 0.38, P = 7e−19) and ImmuneScore (*R* = 0.86, P = 1e−146) (Figure 5C). These results suggested that DEGs of the turquoise modules played an important role in influencing clustering and immune profiles.

### Functional Enrichment Analysis and PPI Network Analysis of Hub Genes

**FIGURE 6:**
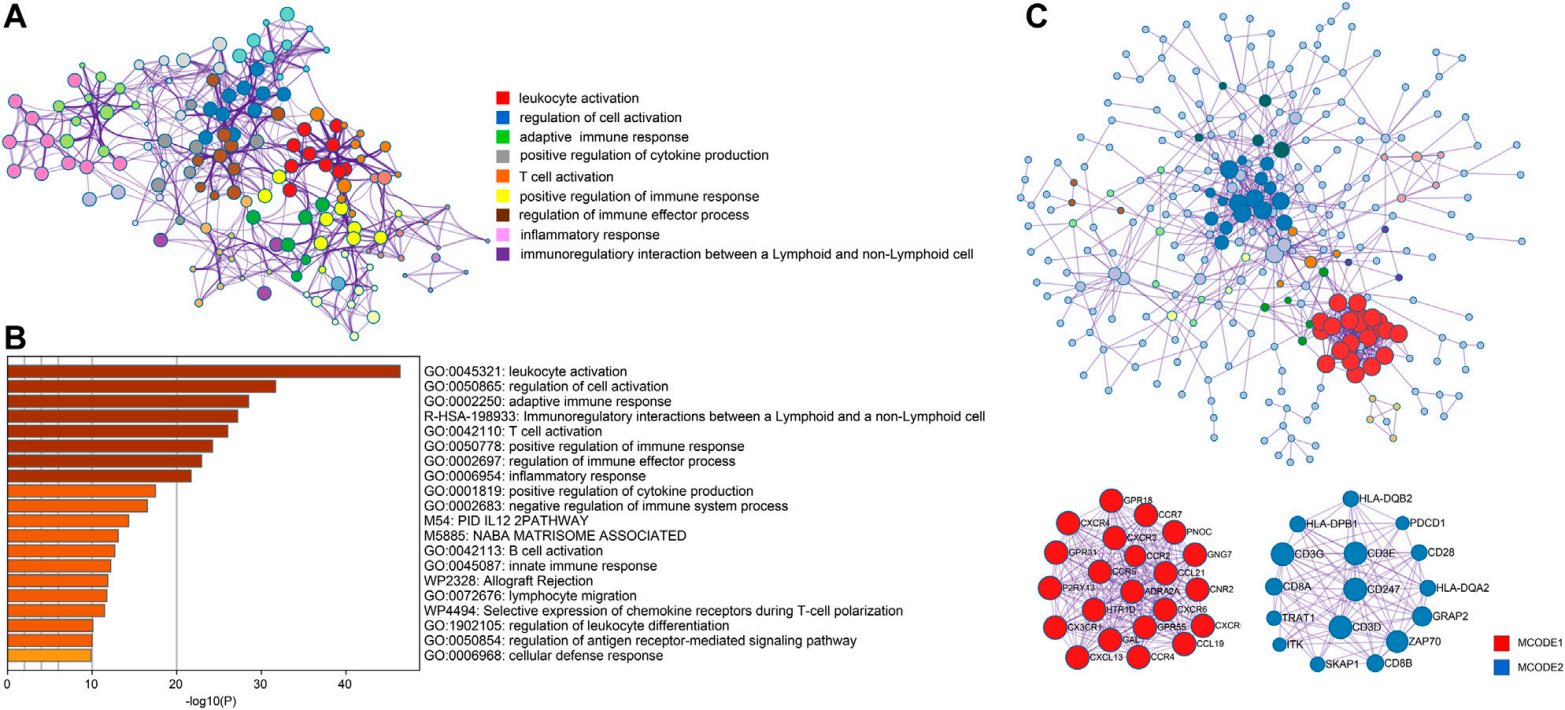
Analysis of functional enrichment and construction of PPI network. **(A)** Selected enriched terms for a network, colored by cluster group ID. **(B)** Functional enrichment analysis in various ontology sources. **(C)** Protein-protein interaction network analysis for whole selected genes and two highlighted MCODE components.

In order to better understand genes in the turquoise model, we uploaded them to Metascape for functional enrichment analysis and constructed the PPI network. The most significant pathways in the functional enrichment analysis were related to immune modulation, including lymphocyte activation, positive regulation of immune response, regulation of immune effector process, and B cell activation (Figures 6A,B). In the PPI network analysis, the MCODE algorithm further divided the whole PPI network into two major MCODEs. The MCODE 1 was related to G alpha (i) signaling events and GPCR ligand binding. The MCODE2 contained 16 genes (*PDCD1*, *CD28*, *CD247*, *CD3D*, *CD3E*, *CD3G*, *CD8A*, *CD8B*, *HLA-DPB1*, *HLA-DQA2*, *HLA-DQB2*, *GRAP2*, *TRAT1*, *SKAP1*, *ZAP70,* and *ITK*) was tightly correlated with the generation of second messenger molecules and T-cell receptor signaling pathway (Figure 6C).

### Verification of Immune Characteristics of Clustering Based on YTHDF1 and IGF2BP2 in the GEO Database

**FIGURE 7:**
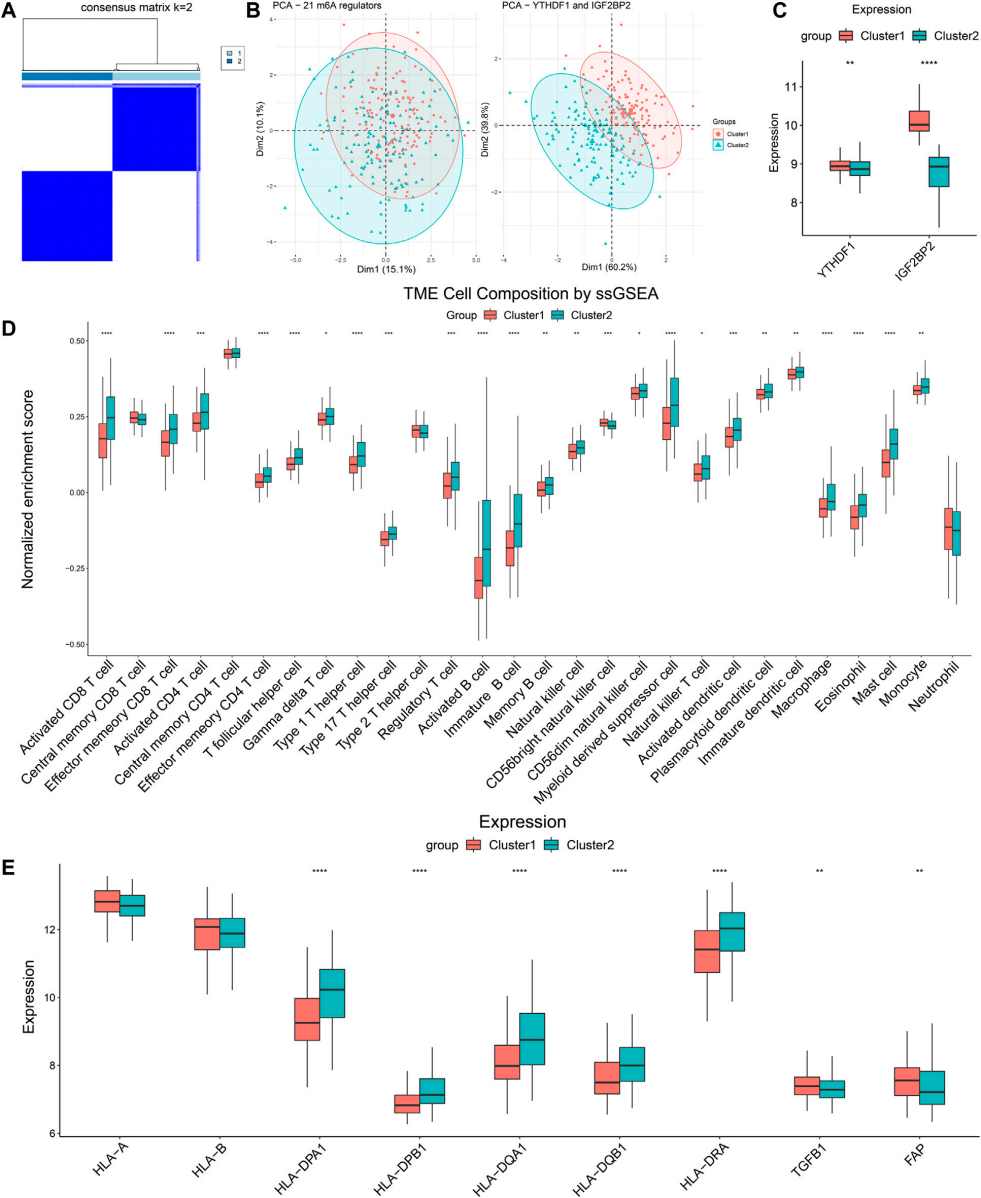
Validation of clustering based on YTHDF1 and IGF2BP2 in GSE65858. **(A)** Consensus clustering matrix for k = 2. **(B)** The results of PCA of clustering based on 21 m6A regulators as well as *YTHDF1* and *IGF2BP2*. **(C)** Comparison of expression level of *YTHDF1* and *IGF2BP2* between Cluster 1 and Cluster 2. **(D)** ssGSEA indicated different immune cells infiltration between two clusters. **(E)** Comparison of immune-related molecules between Cluster 1 and Cluster 2. *p < 0.05, **p < 0.01, ***p < 0.001, ****p < 0.0001.

To externally validate the significance of the m6A reader-based clustering, we obtained the expression profiling data of the GSE65858 array from the GEO database. The GSE65858 array involved 270 patients with HNSCC. Similarly, we performed consensus clustering in the 270 patients using the R package “ConsensusClusterPlus,” and found that the optimal number of clustering was 2 (Figure 7A). PCA plot indicated the above clustering had good efficiency of distinction (Figure 7B). There were 137 patients in Cluster 1 and 133 patients in Cluster 2, and the latter had significantly lower expression of *YTHDF1* and *IGF2BP2* (Figure 7C). We also performed CIBERSORT and ssGSEA to estimate TME composition. The results of CIBERSORT showed that, compared with Cluster 1, Cluster 2 had significantly more plasma cells, CD8^+^ T cells, regulatory T cells, gamma delta T cells, and less activated dendritic cells and activated mast cells (Supplementary Figure S4). In addition, results obtained from ssGSEA indicated that Cluster 2 highly expressed activated CD8^+^ T cells, activated CD4^+^ T cells, T helper cells (Type 1 and 17), and activated B cells, indicating that Cluster 2 had a more favorable TIME than Cluster 1 (Figure 7D). A comparison of immune-related molecules was also performed, and we found Cluster 2 had higher expression of MHC II molecule and lower levels of TGF-β and FAP (Figure 7E). The above results from the GEO database confirmed that *YTHDF1-* and *IGF2BP2*-based patient clustering showed distinct immune profiles.

## Discussion

m6A methylation on mRNA is an abundant internal epigenetic modification that has attracted great attention in recent decades, especially in the tumor research area. Exiting research studies had revealed the complex roles of m6A in cancer by regulating the expression of oncogenes and tumor suppressor genes. This effect was cancer-dependent and also varied among different types of m6A regulators. For instance, the m6A writer, METTL3 was found to promote the translation of c-MYC and BCL2 to accelerate leukemia progression by suppressing differentiation and apoptosis in acute myelocytic leukemia (Vu et al., 2017). On the other hand, the m6A eraser, FTO was found to promote the degradation of BNIP3 and inhibited the proliferation and invasion of breast cancer cells (Niu et al., 2019). And the readers, including EIF3, YTH family, and IGF2BP family, mainly regulated the translation and degradation of targeted RNA to participate in m6A modification. Recent studies demonstrated that m6A modification played an important role in regulating the immune response. Targeting and disabling IGF2BPs through circNDUFB2 could prevent the progression of non-small cell lung cancer and activate anti-tumor immunity (Li et al., 2021). YTHDF1 mediated the increase of lysosomal proteases and tumor antigen degradation in dendritic cells and could weaken anti-tumor response and disable CD8^+^ T cells (Han et al., 2019). However, research about the effect of m6A modification on HNSCC was inadequate.

We utilized transcriptomic data from the TCGA dataset and GEO dataset to establish an m6A regulator-based immune phenotype of HNSCC. Specifically, we found that two readers of m6A, *IGF2BP2*, and *YTHDF1* could effectively indicate immune-stimulatory and immune-suppressive HNSCC. To this end, we scored every patient in the TCGA cohort with the ESTIMATE tool and CIBERSORT algorithm to calculate the correlation of m6A regulators with ESTIMATE scores and immune cells infiltration. The absolute Spearman’s coefficient of ImmuneScore of *IGF2BP1*, *IGF2BP2*, *IGF2BP3* and *YTHDF1* were above 0.2. We selected two with the highest absolute coefficients, *IGF2BP2,* and *YTHDF1* for the following analysis. We found patients with HNSCC could be divided into two clusters with different immune profiles based on the expression of *IGF2BP2* and *YTHDF1*. TME was roughly categorized into three types, namely “infiltrated,” “excluded” and “desert” (Hegde and Chen, 2020). The infiltrated type was characterized by sufficient infiltration of CD8^+^ T cells and a high level of MHC I molecule. Desert type was featured by the absence of CD8^+^ T cell infiltration, low level of MHC I molecule, and high level of FAP. According to the expression profiles of immune cells and immune molecules, we tended to consider Cluster 1 as excluded or desert type and Cluster 2 as infiltrated type. In addition, GSEA demonstrated that a number of signal pathways related to immune response were enriched in Cluster 2. Therefore, it was speculated that the TME of Cluster 2 was more favorable for ICIs-based immunotherapy.

Overexpression of *EGFR* could be detected in over 90% of HNSCC, which was an important signal receptor that brings about tumorigenesis, proliferation, and metastasis through downstream pathways, including PI3K/AKT and MAPK (Nicholson et al., 2001; Citri and Yarden, 2006). CD276, also named B7-H3, was one of the immune checkpoint molecules, which was upregulated in HNSCC and helped tumor cells evade immunological surveillance. High expression of *CD276* was related to the occurrence, progression, and metastasis of HNSCC(Wang et al., 2021). Both *EGFR* and *CD276* were found highly expressed in Cluster 1, indicating that HNSCC of Cluster 1 were more likely to correlate with worse biological behavior, poorer clinical result, and insensitivity to ICIs-based immunotherapy. However, EGFR antibodies or CD276 blockade could be considered for HNSCC of Cluster 1.

To further investigate internal influencing factors between the two clusters, we screened DEGs and performed WGCNA to find the module closely related to clustering and ImmuneScores. Successively, we constructed a PPI network, we finally obtained 16 genes (*PDCD1*, *CD28*, *CD247*, *CD3D*, *CD3E*, *CD3G*, *CD8A*, *CD8B*, *HLA-DPB1*, *HLA-DQA2*, *HLA-DQB2*, *GRAP2*, *TRAT1*, *SKAP1*, *ZAP70,* and *ITK*). These 16 hub genes were all upregulated in Cluster 2. PDCD1 was a receptor of immunosuppression usually expressed in activated T cells. CD28 played an essential role in T cells proliferation, and survival, and provided the second signal for T cell activation (Esensten et al., 2016). CD247, CD3D, CD3E, and CD3G participated in constituting T-cell receptor-CD3 complex (TCR-CD3) to recognize antigens and deliver the first signal for T cell activation (Kuhns et al., 2006). CD8A and CD8B acted as co-receptors for TCR (Rudolph et al., 2006). MHC II molecule was encoded by *HLA-DPB1*, *HLA-DQA2,* and *HLA-DQB2* and played an important role in antigens binding and cross-presentation. Proteins encoded by *ZAP70* and *ITK* belonged to the tyrosine kinase family, which were critical for signal transduction in T cells (Berg, 2007; Au-Yeung et al., 2018). And proteins encoded by *GRAP2*, *TRAT1*, and *SKAP1* also played an important role in signal transduction (Raab et al., 2010). It was, therefore, suggested that most of these DEGs were highly relevant to immune response and might contribute to different immune profiles between the two clusters.

There were several limitations in our study. We performed the study based on TCGA and GEO databases without verification using a clinical dataset. Only correlation analysis on phenotype level was conducted. There lacked a demonstration on the protein level. Last but not least, there was a lack of mechanistic study.

In conclusion, our study divided HNSCC into two clusters based on *IGF2BP2* and *YTHDF1*, which provided a simple and feasible tool to identify HNSCC with different immune profiles and helped estimate sensitivity to ICIs-based immunotherapy. We preliminarily explore the possible mechanisms, combined with the previous research works about IGF2BP2 and YTHDF1, and we speculated that they might hamper the expression of specific genes, which were related to antigen recognition, signal transduction, proliferation, and activation of effector T cells. Meanwhile, they might increase the stability of tumorigenic genes, such as *EGFR* and *CD276*, and excessively activated downstream signal pathways. The joint effect led to different biologic behavior and immune profiles in the two clusters. The existing study had found knocking down *YTHDF1* could enhance the therapeutic efficiency of ICIs in mice (Han et al., 2019). It would be important to determine whether suppressing the expressions of *IGF2BP2* and *YTHDF1* could modulate the sensitivity to ICI-based immunotherapy. Specific molecular mechanisms awaited further exploration.

## Data Availability

The original contributions presented in the study are included in the article/Supplementary Material; further inquiries can be directed to the corresponding author.

## Abbreviations

CPS, combined positive score; DEG, differently expressed gene; EBV, Epstein–Barr virus; FAP, fibroblast activation protein alpha; GEO, the Gene Expression Omnibus; GSEA, gene set enrichment analysis; HNSCC, head and neck squamous cell carcinoma; HPV, human papillomavirus; ICIs, immune checkpoint inhibitors; m6A, N6-Methyladenosine; MCODE, the Molecular Complex Detection; mRNA, messenger RNA; PCA, principal component analysis; PPI, protein-protein interaction; ssGSEA, single sample GSEA; TCGA, The Cancer Genome Atlas; TGF-β, transforming growth factor-β; TIME, tumor immune microenvironment; TME, tumor microenvironment; WGCNA, weighted gene co-expression network analysis.
