# Supplementary material for: Identification of Two m6A Readers YTHDF1 and IGF2BP2 as Immune Biomarkers in Head and Neck Squamous Cell Carcinoma
Source: Front Genet. 2022 May 12;13:903634. doi: 10.3389/fgene.2022.903634 (PMC9133459; doi:10.3389/fgene.2022.903634)
Supplement: Supplementary file 1 [file DataSheet2.docx]

| Gene | Protein | Function |
| --- | --- | --- |
| CD274 | programmed cell death 1 ligand 1 | The encoded protein is a type I transmembrane protein. Interaction of this ligand with its receptor inhibits T-cell activation and cytokine production, which provides an immune escape for tumor cells through cytotoxic T-cell inactivation. |
| CD276 | B7-H3 | highly expresses in HNSCC as a checkpoint, which promotes tumorigenesis, metastasis and evasion of immune surveillance |
| PDCD1 | programmed cell death protein 1 | an immune-inhibitory receptor expressed in activated T cells, which regulates T-cell functions and promotes the differentiation of CD4+ T cells into T regulatory cells |
| CTLA4 | cytotoxic T-lymphocyte protein 4 | a member of the immunoglobulin superfamily, which transmits an inhibitory signal to T cells |
| TNFRSF4 | tumor necrosis factor receptor superfamily member 4 | a receptor for TNFSF4/OX40L/GP34, which is a costimulatory molecule implicated in long-term T-cell immunity |
| EGFR | epidermal growth factor receptor | a transmembrane glycoprotein that is a member of the protein kinase superfamily, which leads to tumor cell proliferation and invasion |
